# Supplementary material for: Seroprevalence of SARS-CoV-2 specific IgG antibodies in District Srinagar, northern India – A cross-sectional study
Source: PLoS One. 2020 Nov 11;15(11):e0239303. doi: 10.1371/journal.pone.0239303 (PMC7657487; doi:10.1371/journal.pone.0239303)
Supplement: Attachment — Submitted filename: Response to Reviewers.docx [file pone.0239303.s006.docx]

**Editor-in-Chief**

**PLOS ONE**

I am submitting the revised manuscript for the article # PONE-D-20-27283 titled “Seroprevalence of SARS-CoV-2 specific IgG antibodies in District Srinagar, northern India– a cross-sectional study”.

We have made necessary changes in the manuscript taking into consideration the recommendations of the reviewers. Responses to reviewer comments and changes made in the manuscript are enlisted below.

| **Reviewer No** | **Comments** | **Response to comments** | **Changes made in the manuscript** |
| --- | --- | --- | --- |
| 1 | I am not familiar with "cold chain" and you may want to define "test med", procedure. | Cold chain is a system used for transportation of vaccines under appropriate temperature conditions. We used a similar kind of transportation system for the blood/serum samples.  The “test med” was a typographical error. Thanks for pointing it out. | Line 120-122 now reads *“Centrifuged blood samples were transported under the cold chain (vaccine carrier with ice packs) to a central laboratory for further processing and testing.”* instead of the previous *“Centrifuged blood samples were transported under the cold chain to a central laboratory for further processing and testing”*.  Line 125 now reads *“We performed the test using …..*” instead of the previous “*We performed the test med using …..*” |
| 1 | What was the performed RT-PCR sensitivity/specificity? | The RT-PCR was performed by independent diagnostic laboratories and they use a variety of kits for diagnosis of SARS-CoV-2 infection. The sensitivity/specificity of these kits varies to some extent. It is not possible for us to comment on the sensitivity/specificity of RT-PCR, because it is impossible to link the test kit to a specific patient. | No changes made. |
| 2 | Knowing the time between the onset of symptoms and your serology test would be helpful in light of the lack or post-infection immunity for discussion and future reference/research. | Unfortunately, we did not obtain data about date of onset of symptoms. We only enquired about the presence of any symptoms in the four weeks before the interview. We appreciate your suggestion and are working on obtaining such information in a different set of patients. Thanks for the comment. | No changes made. |
| 2 | Very nice article. Well written. Would like to know how they did the adjustment for specificity of the test in  getting their overall positivity rate rather than just seeing the reference. Also, would like to see how the test performed in  individuals who had been RT-PCR positive. I can’t find that in the primary paper. Also, I would prefer to see the methods you  used to adjust for test specificity rather than just seeing the reference. | Thank you for your comments. We have added the formula used in the manuscript even though it also appears in the Supplementary File (S2 Table).  There were only 8 RT-PCR positive individuals in our study. Out of them 5 were positive for SARS-CoV-2 specific IgG antibodies. We didn’t include this information in the primary paper because of the small number of such patients given that it would not have been proper to make inferences and conclusions based on such small numbers. We also did not have information about the timing of the RT-PCR which again made making any inferences/conclusions difficult. | Line 206-209 now reads “*When adjusted for sensitivity and specificity of the laboratory test kit [14], the number of infections comes down to 32602 (95% CI 25470-39734) (S2 Table). [Prevalence adjusted for sensitivity and specificity of the test was calculated as: (Age- and gender-adjusted prevalence + Test specificity – 1) ÷ (Test sensitivity + Test specificity – 1)].*” Instead of the previous “*When adjusted for sensitivity and specificity of the laboratory test kit [14], the number of infections comes down to 32602 (95% CI 25470-39734) (S2 Table).*” |
